# Transforming teaching: wellbeing and professional practice

**DOI:** 10.3389/fpsyg.2023.1192148

**Published:** 2023-06-16

**Authors:** Zhengxi Liu

**Affiliations:** ^1^School of Foreign Languages, Wuhan University of Science and Technology, Wuhan, China; ^2^Faculty of Education, University of Windsor, Windsor, ON, Canada

**Keywords:** teacher education, teacher wellbeing, teacher professional practice, wellbeing education, educational leadership, appreciative inquiry

As an increasing number of countries are experiencing escalating rates of teacher attrition, especially among early career teachers, attention to teacher wellbeing has become a critical factor in attracting and retaining qualified teachers (Craig, 2017; Viac and Fraser, 2020). Under the circumstances, a plethora of factors with positive effects have been identified to enhance teacher wellbeing. However, a more holistic approach that activates the dynamic interplay of practice, research, and policy aiming at teacher wellbeing and professional practice is more than ever needed given the ongoing disruption caused by the pandemic to education (White and McCallum, 2020a,b). Some issues warrant further examinations. For example, how can we integrate wellbeing education into teachers' professional development programs, particularly initial teacher training programs, to prepare them to be both happy and effective teachers? More specifically, how to integrate strength-based and appreciative inquiry approaches to wellbeing education and teaching and learning in professional practice? To address these issues, the book under review, i.e., *Transforming Teaching: Wellbeing and Professional Practice* (White and McCallum, 2022) examines scenarios of wellbeing education in different contexts to support teachers to integrate evidence-informed wellbeing approaches into their professional practice so as to achieve the ultimate goal of education: flourishing and academic growth.

This book consists of eight chapters. Chapter one introduces an overarching conceptual framework that situates and guides the research on teacher wellbeing in each chapter of the book and its linkage to quality teacher education. As the core concept of the framework, teacher wellbeing is defined around five dimensions: social, physical, cognitive, emotional, and spiritual.

In chapter two, drawing on the theory of character strengths (Peterson and Seligman, 2004) and the conceptual model for the positive humanities (Tay and Pawelski, 2022), the author intends to integrate character strengths into the teaching of literature curriculum as a pathway to foster wellbeing in the classroom. Specifically, a two-step strategy was developed to integrate strengths in literature classes. Step one is to ask students to reflect upon and seek examples of each strength in their lives by means of class discussions and writing tasks to promote their understanding of the core characteristics of the strengths. Step two is to encourage students to link their strengths with their own interpretation of the literary texts, characterization, and plot development. This strength-based reflective teaching model connects students' strengths with the strengths demonstrated by the characters in the literature and thus bridges the gap between wellbeing education and strength-based professional practices.

In chapter three, focusing on discovering the best of what is, the author conducted an appreciative inquiry case study of 55 Australian preservice teachers (PST)' perceptions of teaching during COVID-19 and sorted out two overarching themes, namely, PSTs' values and strengths. PSTs' values focused on their judgement belief of what was important for their teaching placement. Flexibility is valued the most across all PSTs' responses as they experienced the constant shifts between in-person and online learning during the pandemic. PSTs' strengths focused on the positive traits that they naturally displayed in the face of challenges and adversity. Resilience is proved to be an integral part of the progression of a PST's professional identity. The findings highlight the importance of the integration of wellbeing into the development of PSTs' professional practices.

In the fourth chapter, the author aims to investigate the links between teacher wellbeing and belonging through a case study of an Australian school that has measured its employee wellbeing twice over a period of 3 years. The results indicate that “teachers' wellbeing is optimal when they have a sense of belonging to their school, are supported by leadership, are resourced to fulfill cognitive needs, and have a collegial climate in which to function” (White and McCallum, 2022, p. 68). Moreover, a higher sense of belonging results in not only teachers' stronger professional identity, job satisfaction and commitment, and teaching effectiveness but also students' better academic achievement.

Chapter five is centered on exploring teachers' wellbeing and their reflections in three areas during the pandemic, including the impact on teaching and learning, changes to teachers' work, and education transformation. With a methodology of appreciative inquiry that focuses on participants' strengths, this study, first, has found that teachers adapt and respond to the impacts on their teaching quickly, students demonstrate more appreciation for schooling, and efforts of school leaders are increasingly recognized as a strong sense of belonging developed in the whole-school community. Then, the study shows that even though the use of technology has made teaching practices more flexible, face-to-face interactions cannot ever be replaced with teacher-student relationships at the core, not the technology. Meanwhile, the study also shows that the pandemic has transformed education so much that it requires every member of the community to work more collectively to respond to the challenges ahead. In brief, teachers are the most critical member in the school community, and their work “should be accepted, rewarded, and valued” (White and McCallum, 2022, p. 88).

Chapter six explores the possibility of integrating appreciative inquiry (AI) 4-D cycle–discovery, dream, design, and destiny–into the strategic planning summit of a disadvantaged school in South Australia. During the one-day summit, the author, working with the school leadership team, operationalized the first three stages of the cycle and discussed the outcomes. In the discovery stage, four rounds of appreciative questions are developed to guide the participants to recount the stories that are symbolic of the school's strengths, through which the core of the school values are generated. In the dream stage, participants are invited to give examples and stories of what an ideal school looks like with the implementation of the new strategic plan. In the design stage, participants are encouraged to introduce strategic initiatives and measures of success for the school to make their vision a reality. The successful application of AI in the strategic planning of a disadvantaged school provides implications for other educational institutions with limited resources and diverse populations to approach similar challenges.

Chapter seven draws on three case studies to examine how quality leadership can facilitate teacher wellbeing and teaching performance. In the first case, the author conducted a comparative study with a sample of 67 early career teachers from both Australia and the UK and found that supportive school leadership impacts teacher wellbeing positively. In the second case, the author conducted an appreciative study involving 770 participants from Australia and found that teachers demonstrate higher levels of wellbeing and sustained career when they feel wellbeing is prioritized at school. In the last case, with 322 respondents from 12 countries, the author discusses how school leaders influence teachers' experience of wellbeing during the pandemic. In a word, school leaders have an important role to play in supporting and nurturing teachers' wellbeing in the workplace.

In the last chapter, the author synthesizes the studies presented in the previous chapters and proposes the potential convergence of wellbeing education and teachers' professional practice to achieve the dual goal of education: academic growth and wellbeing. Moreover, several research priorities for the next decade are provided.

Teacher wellbeing matters. This is the most important message that the book under review wants to convey to the readers. It matters because “teachers are the most important in-school factor influencing student achievement, happiness, and satisfaction and are also pivotal members of the school community” (White and McCallum, 2022, p. 33). This view has significant implications for school leadership, initial teacher education, and education research in the coming decades. To begin with, teacher wellbeing and teaching effectiveness can be significantly advanced through quality school leadership. As the world recovers gradually from the pandemic, teachers are experiencing increasing demands and complexity of their work. The extent of support and resources they receive from the school will be a critical determinant that impacts their satisfaction and engagement with the role. Positive and supportive school leadership will lead the school to become a whole professional learning community and promote a better sense of wellbeing for all. Furthermore, initial teacher education is critical to ensure that graduate teachers are classroom ready and stay in the profession. However, up to 50% of new teachers leave or have the intention to leave the profession within the first 5 years because they are overwhelmed with the challenges of their roles. According to White and McCallum (2020a,b), integrating wellbeing education into initial teacher education programs is an effective response because teacher wellbeing itself is of great importance for “the attraction, retention, and sustainability of the profession” (p. 118). Finally, the “teacher wellbeing matters” message also provides the directions for future education research, that is, integrating wellbeing education into the whole education systems involving school leadership, wellbeing for teachers, wellbeing for students, and community issues. Only then will we “realize the promise of wellbeing education and its potential to transform teaching” (White and McCallum, 2022, p. ix).

Besides, the book can also be taken for researchers and education practitioners as a guide of methodologies to conducting wellbeing research, to name a few, strength-based interventions, appreciative inquiry, and case study. With these methodologies at hand, they would have significant opportunities to integrate wellbeing education with their professional practices more consistently.

Admittedly, it would be more comprehensive if the book could include more participants from different countries other than Australia and the UK, especially from different cultures because wellbeing is conceptualized differently in different cultures (Hine et al., 2022). For example, eastern cultures tend to associate interdependence as important for wellbeing, while western cultures may place a higher value on independence. The inclusion of participants from both western and non-western cultures will make the case studies in the book applicable to many more global contexts.

All in all, a concern should not obscure the good. We believe that the value of the book will not only benefit the whole school community involving school leaders, initial teacher educators, teaching practitioners, and the learners specifically but benefit also the whole society at large, which might build a synergy to transform teaching and learning better for the 21st century.

## Author contributions

The author confirms being the sole contributor of this work and has approved it for publication.
